# Health equity in COVID-19 testing among patients of a large national pharmacy chain

**DOI:** 10.3389/fpubh.2024.1422914

**Published:** 2024-09-11

**Authors:** Tanya Singh, Renae L. Smith-Ray, Elijah Ogunkoya, Amy Shah, Daniel A. Harris, Kaleen N. Hayes, Vincent Mor

**Affiliations:** ^1^Walgreen Co, Deerfield, IL, United States; ^2^Department of Health Services, Policy, and Practice, Brown University School of Public Health, Providence, RI, United States; ^3^Center for Gerontology and Healthcare Research, Brown University School of Public Health, Providence, RI, United States; ^4^Providence Medical Center Veterans Administration Research Service, Providence, RI, United States

**Keywords:** COVID-19, health equity, pharmacy, whole genome sequencing, testing

## Abstract

**Background:**

Several social determinants of health and other structural factors drive racial and ethnic disparities in COVID-19 risk, morbidity, and mortality. Public-private collaborations with community pharmacies have been successful in expanding access to COVID-19 testing and reaching historically underserved communities. The objectives of this study were to describe individuals who sought testing for COVID-19 at a national community pharmacy chain and to understand potential racial and ethnic inequities in testing access, positivity, and infection with emerging variants of concern.

**Methods:**

We conducted a cross-sectional study of individuals aged ≥18 who were tested for COVID-19 (SARS-CoV-2) at a Walgreens pharmacy or Walgreen-affiliated mass testing site between May 1, 2021 and February 28, 2022. Positivity was defined as the proportion of positive tests among all administered tests. A geographically balanced random subset of positive tests underwent whole genome sequencing to identify specific viral variants (alpha, delta, and omicron). Logistic regression estimated odds ratios (ORs) and 95% confidence intervals (CIs) to compare the likelihood of testing positive and testing positive with an emerging variant of concern across race and ethnicity groups.

**Results:**

A total of 18,576,360 tests were analyzed (16.0% tests were positive for COVID-19; 59.5% of tests were from White individuals and 13.1% were from Black individuals). American Indian or Alaska Native (OR = 1.12; 95%CI = 1.10–1.13), Hispanic or Latino (1.20; 95%CI = 1.120, 1.21), and Black (1.12; 95%CI = 1.12, 1.13) individuals were more likely to test positive for COVID-19 compared to White individuals. Non-White individuals were also more likely to test positive for emerging variants of concern (e.g., Black individuals were 3.34 (95%CI = 3.14–3.56) times more likely to test positive for omicron compared to White individuals during the transition period from delta to omicron).

**Discussion:**

Using a national database of testing data, we found racial and ethnic differences in the likelihood of testing positive for COVID-19 and testing positive for emerging viral strains. These results demonstrate the feasibility of public-private collaborations with local pharmacies and pharmacy chains to support pandemic response and reach harder to reach populations with important health services.

## 1 Introduction

SARS-CoV-2 emerged in late 2019 and spread globally, disproportionately affecting racial and ethnic minority groups across the United States (US) and putting them at increased risk of infection, hospitalization, and death (1–5). Between February and July of 2020, Black and Hispanic Americans were nearly 3 to 4 times more likely to die from COVID-19 relative to White Americans, respectively (6). Several social determinants of health and structural factors, such as poverty, crowded housing, and systemic racism drive racial and ethnic inequities in COVID-19 outcomes (7, 8).

Timely and equitable access to SARS-CoV-2 testing provides important individual-level information, such as whether to self-isolate, and community-level information on disease trends and outbreaks. Thus, disparities in SARS-CoV-2 testing can exacerbate existing inequities in SARS-CoV-2 infection and its sequelae. The CDC’s Increasing Community Access to Testing, or ICATT program [formerly known as Community-Based Testing Sites (CBTS)] was established to improve access to COVID-19 testing by collaborating with a range of private and community partners and covering the cost of testing for uninsured patients. Pharmacies and large pharmacy chains were important partners in the program because they are distributed widely throughout the US, most Americans live close to a pharmacy (9), and pharmacies have trained staff that provide other public health services, such as vaccinations (10). The ICATT program also enabled the creation of national repositories of testing data to examine testing equity and to monitor the distribution of emerging SARS-CoV-2 variants. Although prior studies have examined testing equity and racial and ethnic variation in the burden of novel/emerging variants of concern (11, 12), few have leveraged nationally representative data following the widespread adoption of CBTS and ICATT. Investigations that use testing data from large pharmacy chains will be useful for demonstrating the capacity for pharmacies to contribute to public health research and monitoring, evaluating SARS-CoV-2 testing equity, and documenting variation in the burden of specific viral variants over time, especially for harder to reach populations.

Using national testing data from Walgreens pharmacies, our objectives were to describe individuals who were tested for COVID-19 and to understand potential racial and ethnic inequities in testing access and positivity. Among those who tested positive for SARS-CoV-2, we also examined racial and ethnic differences in the burden of novel SARS-CoV-2 variants using whole genome sequencing.

## 2 Methods

### 2.1 Study design, data sources, and population

We conducted a cross-sectional study of patients aged ≥18 who underwent testing for SARS-CoV-2 at a Walgreens pharmacy or Walgreens-affiliated mass testing site between May 1, 2021 (first date data was available for analysis) and February 28, 2022 (when the Omicron variant represented >99% of cases). Prior to testing, all patients were asked to complete an online pre-screening clinical questionnaire (see section 3.3). Patients’ SARS-CoV-2 testing results and clinical data were stored in a secure computing environment at Walgreens.

### 2.2 SARS-CoV-2 testing and genomic analysis

SARS-CoV-2 tests were administered via nasal self-swabs, with instructions and processing by trained pharmacy or clinical staff. Real-time reverse transcription–polymerase chain reaction (RT-PCR), nucleic acid amplification test (NAAT), and rapid antigen SARS-CoV-2 tests were performed during the study period. Rapid tests were processed at the pharmacy and results were provided to the patient within 1 h. Non-rapid swabs were processed for SARS-CoV-2 at external lab facilities and results were reported to patients within approximately 48 h. All test results were categorized into a binary outcome variable of positive vs. not positive for SARS-CoV-2.

A random subset of positive RT-PCR test results for SARS-CoV-2 underwent genomic sequencing to understand changes in the temporal, geographic, and sociodemographic distribution of SARS-CoV-2 variants (Alpha, Delta, and Omicron strains). Genomic sequencing was performed by Aegis Sciences Corporation laboratory in Nashville, Tennessee. RT-PCR positive specimens with sufficient quality genetic material [average cycle threshold (Ct) value <30] qualified for genome sequencing, with a subsequent stratified randomization (based on geographic testing volume) to select a regionally representative sample of positive tests.

### 2.3 Clinical and sociodemographic pre-screening data

The pre-screening questionnaire collected patients’ age, gender, race, ethnicity, address, vaccination history, SARS-CoV-2 exposure history, and current symptoms. Self-reported race categories included: American Indian or Alaskan Native, Asian, Black or African American, Native Hawaiian or other Pacific Islander, and White. Ethnicity was limited to Hispanic or Latino or non-Hispanic or Latino. We categorized all Hispanic or Latino patients in one category, with all other race and ethnicity categories being non-Hispanic or Latino. We also recorded information to describe the SARS-CoV-2 testing sites, including population density and rurality (rural, suburban, and urban) and median household income at the zip code level.

To understand testing equity, we compared the distribution of median household income and different racial and ethnic groups in our testing population to nationally representative samples from the 2020 American Community Survey (ASC) (13).

### 2.4 Statistical analysis

We conducted descriptive univariable and bivariable statistics to describe differences in the distribution of testing and test positivity over time and by patients’ sociodemographic characteristics. Unadjusted odds ratios were calculated using logistic regression to compare crude associations between race and ethnicity and testing positive for COVID-19. Next, we calculated adjusted odds ratios, adding the following covariates to the model: patient age, gender, income, state of residence, and testing calendar week. Covariates were maintained in the model if *p* < 0.05.

For the subset of positive tests that underwent genomic sequencing, we analyzed two transition periods: (1) the Alpha to Delta variant of concern transition (May 23, 2021-July 17, 2021) and (2) the Delta to Omicron variant of concern transition (December 5, 2021-January 1, 2022). Variant of concern transition periods were defined according to changes in prevalence of specific variants over time, starting once a new variant represented 5% of positive tests and ending once its prevalence reached 90% of positive tests. The outcome for this analysis was testing positive for the emerging variant vs. testing positive for any other variant. Due to the smaller population in this portion of the study, patients who identified as American Indian or Alaska Native, Native Hawaiian or Other Pacific Islander were categorized as ‘other race and ethnicity’, along with those who declined to provide their race and ethnicity. Logistic regression compared the likelihood of testing positive for an emerging variant (e.g., omicron) vs. existing variant (e.g., delta) during transition periods across different races and ethnicities.

In secondary analyses, we examined whether racial and ethnic differences in the risk of emerging SARs-CoV-2 variants were modified by rural/urban geography. The unadjusted odds of testing positive for the Delta and Omicron variants were compared across racial and ethnic groups and stratified by rural/urban geography.

All data analyses were performed using SAS Enterprise Guide version 7.1 (Cary, NC). As a descriptive epidemiologic study, traditional hypothesis testing was not used.

## 3 Results

Data were gathered from 5,198 testing sites in 49 US states, the District of Columbia, and Puerto Rico. A total of 18,790,514 tests were identified for study inclusion. We excluded 214,154 tests with invalid results due to insufficient specimen amounts for processing. The final analytic sample represented 18,576,360 tests. Among 18,576,360 tests, 16.0% tests were positive for SARS-CoV-2. A total of 59.5% of tests were collected from White patients, 56.4% from women, 60.7% of tests were completed in a suburban pharmacy, median income (based on pharmacy zip code) was $70,081, and the mean age was 36.3 years (Table 1). The Walgreens testing population was generally representative of the overall U.S. racial and ethnic composition according to the 2020 ACS 5-year US estimates for Black or African American (13.1% vs. U.S. estimate of 12.6%), White (59.5% vs. 60.6%), and Hispanic or Latino patients (18.2% vs. 19.6%). However, the Walgreens testing population was more female (U.S. 50.4%), younger (U.S. 38.8 years), and received testing in areas with slightly higher median income (U.S. median $67,521) than ACS estimates.

**Table 1:** Demographics of testing population by test result and positive tests with whole genome sequencing (WGS).

	Total, n (column%)	SARS-CoV-2 positive, n (row%)	SARS-CoV-2 negative, n (row%)	Positive tests with WGS, n (column%)
Total	18,576,360 (100.0)	2,971,626 (16.0)	15,604,734 (84.0)	318,196 (100%)
Gender				
Male or female	18,524,750 (99.7)	2,965,203 (16.0)	15,559,547 (84.0)	317,537 (99.8)
Male	8,070,875 (43.6)	1,396,980 (17.3)	6,673,895 (82.7)	153,210 (48.2)
Female	10,453,875 (56.4)	1,568,223 (15.0)	8,885,652 (85.0)	164,327 (51.8)
Other	51,610 (0.3)	6,423 (12.4)	45,187 (87.6)	659 (0.2)
Median household income, mean (SD)	$70,081 ($25,706)	$66,595 ($23,785)	$70,744 ($25,991)	$68,332 ($24,412)
Age, mean (SD)	36.3 ± 18.4	35.7 ± 17.7	36.4 ± 18.5	35.4 ± 17.3
Race/ethnicity				
Answered	17,242,582 (92.8)	2,779,340 (16.1)	14,463,242 (83.9)	296,349 (93.1)
American Indian or Alaskan Native	101,639 (0.6)	17,806 (17.5)	83,833 (82.5)	1,640 (0.6)
Asian	1,353,312 (7.8)	144,621 (10.7)	1,208,691 (89.3)	16,775 (5.7)
Black or African American	2,262,739 (13.1)	394,031 (17.4)	1,868,708 (82.6)	41,476 (14.0)
Hispanic or Latino	3,141,614 (18.2)	569,305 (18.1)	2,572,309 (81.9)	59,330 (20.0)
Native Hawaiian or Other Pacific Islander	127,396 (0.7)	15,084 (11.8)	112,312 (88.2)	1,944 (0.7)
White	10,255,882 (59.5)	1,638,493 (16.0)	8,617,389 (84.0)	175,184 (59.1)
Decline to Answer	1,333,778 (7.2)	192,286 (14.4)	1,141,492 (85.6)	21,847 (6.9)
Urbanicity				
Rural	4,455,325 (24.0)	835,027 (18.7)	3,620,298 (81.3)	89,277 (28.1)
Suburban	11,280,315 (60.7)	1,777,032 (15.8)	9,503,283 (84.2)	206,281 (64.8)
Urban	2,840,719 (15.3)	359,567 (12.7)	2,481,153 (87.3)	22,638 (7.1)

**Figure 1:**
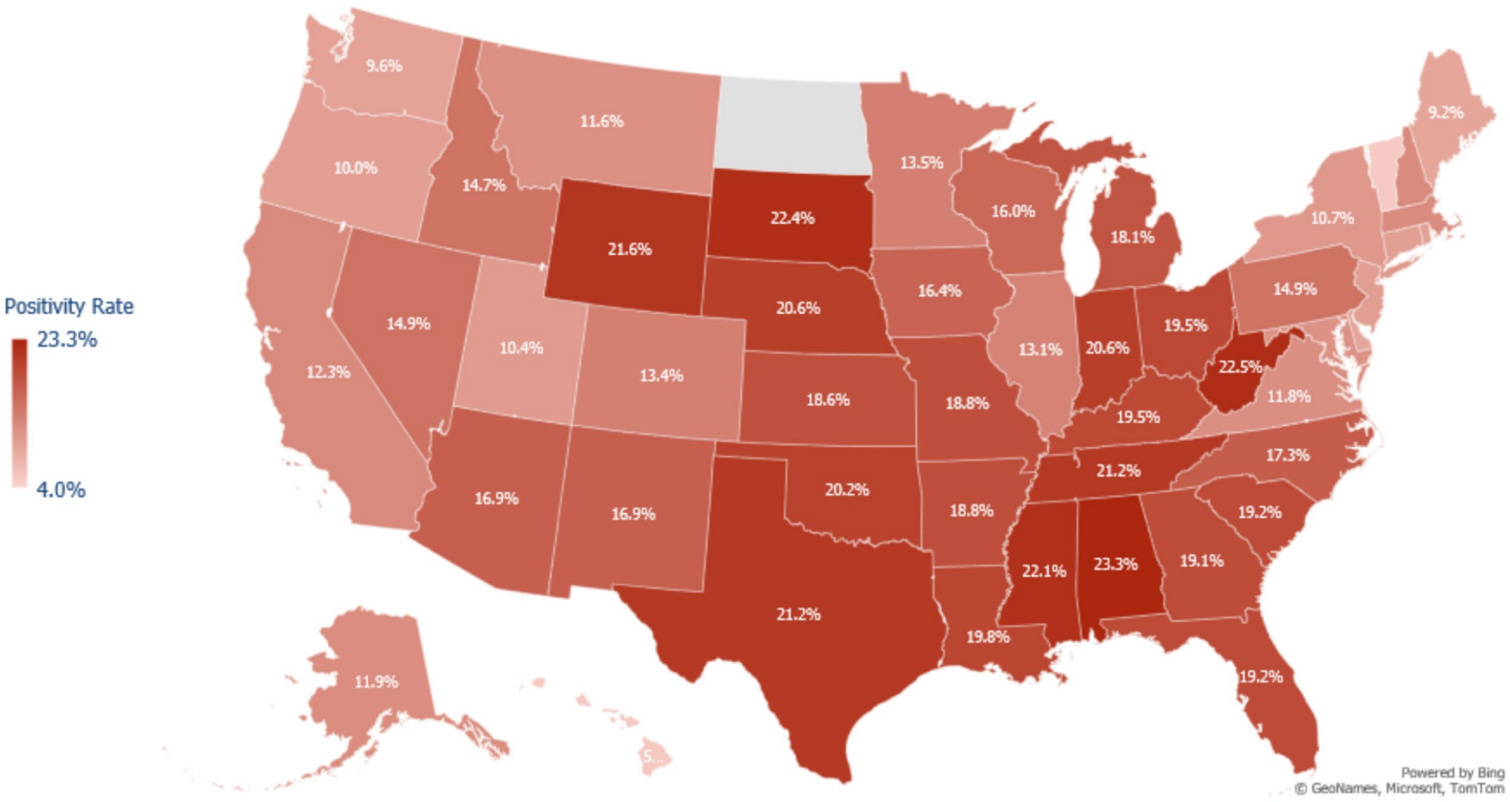
Map of COVID-19 test positivity rate in the United States among adults testing for COVID-19 at a Walgreens pharmacy or Walgreen-affiliated mass testing site between May 2021 to February 2022. Positivity was calculated as the number of positive tests for SARS-CoV-2 divided by the total number of tests administered in each state during the study period. Northeast States Positivity Rate = CT: 10.0%, DE: 9.2%, MA: 12.0%, MD: 11.4%, ME: 9.2%, NH: 12.1%, NJ: 10.1%, NY: 10.7%, PA: 14.9%, RI: 9.8%, VT: 5.3%. Walgreens does not have stores in North Dakota, so there is no positivity estimate for this state.

Over the course of the study, tests conducted in the South and Midwest regions had higher positivity rates than those in the Northeast and the West regions (Figure 1). Asian and Native Hawaiian or other Pacific Islander individuals had reduced odds of testing positive compared to White individuals (adjusted OR 0.652 [95% CI = 0.648–0.656] and 0.894 [95% CI = 0.878–0.910] respectively). American Indian or Alaska Native and Hispanic or Latino individuals had increased odds of testing positive compared to White individuals (adjusted OR 1.072 [95% CI = 1.054–1.090] and 1.109 [95% CI = 1.105–1.113]). Unadjusted, Black or African American patients had increased odds of testing positive compared to White patients (1.124 [95% CI = 1.120–1.128]); however, after adjustment, these patients were at slightly decreased odds of testing positive (0.976 [95% CI = 0.972–0.980]) (Table 2).

**Table 2:** Multivariable logistic regression model of SARS-CoV-2 positivity by race and ethnicity.

Race and ethnicity	Unadjusted OR (95% CI)	Adjusted OR (95% CI)
American Indian or Alaskan Native	1.116 (1.098, 1.134)	1.072 (1.054,1.090)
Asian	0.609 (0.606, 0.613)	0.652 (0.648, 0.656)
Black or African American	1.124 (1.120, 1.128)	0.976 (0.972, 0.980)
Hispanic or Latino	1.201 (1.197, 1.205)	1.109 (1.105, 1.113)
Native Hawaiian or other Pacific Islander	0.704 (0.692, 0.716)	0.894 (0.878, 0.910)
White	Ref	Ref
Decline to Answer	0.877 (0.872, 0.881)	0.936 (0.931, 0.941)

Model is adjusted for age, gender, income, state, and calendar week.

### 3.1 Whole genome sequencing positive results

Between May 1, 2021 and February 28, 2022, 318,196 (10.7% of positive RT-PCR tests) were successfully sequenced, identifying 378 unique genomic lineages. Test results were 51.8% female, 59.1% White, 64.8% suburban, in zip codes averaging an income of $68,332, with a mean age of 35.4 years (Table 1).

Compared to the overall positive cases (*N* = 2,971,626), the subset of sequenced positives was similar to all positives across racial and ethnic groups: 59.1 and 59.0% among white individuals, 20.0 and 20.5% for Hispanic or Latino individuals, 14.0 and 14.2% for Black or African American individuals, and 5.7 and 5.2% for Asian individuals. Median income (based on pharmacy zip code) was also similar ($66,595 for all positive cases) (Table 1).

During the overall study period, 2.5% of the patient population tested positive for Alpha, 70.5% for Delta, and 23.2% for Omicron. The remaining 3.9% of the study population tested positive for other variants, such as Beta, Epsilon, Eta, Gamma, Iota, Kappa, Lambda, and lineages that were not categorized.

Compared to White patients, the unadjusted analysis demonstrated that Asian patients had increased odds of testing positive for Delta (OR = 1.38 [95% CI =1.20–1.69]), Black or African American patients had no difference (OR = 1.00 [95% CI =0.89–1.23]), and Hispanic or Latino patients had decreased odds (OR = 0.82 [95% CI = 0.75–0.91]) whereas the unadjusted odds of testing positive for Omicron were higher for all racial and ethnic groups examined relative to White patients (Table 3).

**Table 3:** Multivariable logistic regression models of testing positive for the emerging variant during two transition periods.

Race/Ethnicity	Unadjusted OR (95% CI) Total N	Adjusted analysis		
		Rural OR (95% CI) *n*	Suburban OR (95% CI) *n*	Urban OR (95% CI) *n*
Delta transition period (May 23, 2021-July 17, 2021)				
Asian	1.375 (1.119–1.689) *453*	1.376 (0.615–3.082) *40*	1.048 (0.752–1.460) *293*	0.793 (0.438–1.438) *120*
Black or African American	1.002 (0.893–1.126) *1,669*	1.326 (0.817–2.151) *159*	0.938 (0.778–1.130) *1,182*	0.784 (0.511–1.204) *328*
Hispanic or Latino	0.824 (0.748–0.908) *2,817*	1.044 (0.741–1.471) *300*	0.732 (0.627–0.856) *1.912*	0.445 (0.308–0.644) *605*
White	Ref	Ref	Ref	Ref
Other	0.945 (0.828–1.078) *1,179*	0.957 (0.636–1.439) *194*	0.710 (0.575–0.876) *760*	0.743 (0.462–1.196) *225*
Omicron transition period (December 5, 2021-January 1, 2022)				
Asian	2.358 (2.161–2.572) *2,642*	1.642 (1.209–2.229) *317*	1.616 (1.421–1.836) *1,989*	0.948 (0.661–1.359) *336*
Black or African American	3.341 (3.135–3.560) *6,406*	3.097 (2.490–3.853) *1,005*	2.561 (2.330–2.815) *4,874*	1.289 (0.931–1.787) *527*
Hispanic or Latino	2.087 (1.973–2.208) *7,191*	1.439 (1.176–1.761) *1,018*	1.065 (0.969–1.170) *5,007*	0.527 (0.405–0.686) *1,166*
White	Ref	Ref	Ref	Ref
Other	1.330 (1.229–1.439) *2,848*	1.274 (1.017–1.596) *643*	1.142 (1.010–1.290) *1,912*	0.988 (0.680–1.437) *293*

Model is adjusted for age, gender, income, state, and calendar week.

### 3.2 Secondary analyses

Rural/urban geography modified the association between race and ethnicity and type odds of emerging SARs-CoV-2 variant (Table 3). In rural communities, Asian, Black, and African American patients had increased odds of testing positive for an emerging variant, especially the Omicron variant (e.g., odds of testing positive for Omicron; Black or African American: OR = 3.10 [95% CI = 2.49–3.85]); Asian: (OR = 1.64 [95% CI = 1.21–2.23]) and Hispanic or Latino (OR = 1.44 [95% CI = 1.18–1.76]).

## 4 Discussion

Using a large, national sample of SARS-CoV-2 testing data collected across US pharmacy locations, we examined the associations between positivity and variants of concern with race and ethnicity. The Walgreens testing population was generally similar to the overall U.S. population in terms of income and race and ethnicity. Examining test results, we found that non-White racial and ethnic groups were generally more likely to test positive for COVID-19 relative to their White counterparts. Additionally, non-White individuals were more likely to test positive for Omicron as a variant of concern relative to White individuals, and degree of urbanicity modified this strength of this relationship.

To examine the capacity of the CBTS and ICATT programs to reach racial and ethnic minorities and other hard to reach groups, we compared Walgreens testing data to US Census estimates. Although we found the testing population to be 2.5 years younger and more female, income (based on pharmacy zip code) was only slightly higher (3.8%), and the population was analogous in terms of race and ethnicity (all race and ethnicity categories were within 1.5 percentage points compared to U.S. population estimates). Among positive tests, WGS was performed similarly across the sociodemographics of gender, age, race and ethnicity, and income, however, it differed by population density of the testing locations. Suburban locations had the highest percent of positive test results which were sequenced, followed by rural, and urban (12, 11, and 6%, respectively). These results demonstrate the feasibility of public-private collaborations with local pharmacies and pharmacy chains to support pandemic response and reach harder to reach populations with important health services.

Regarding positivity, higher rates were observed among tests completed in rural testing locations, the Southern and Midwestern states, lower income communities, among male patients, younger patients, and American Indian or Alaska Native, Black or African American, and Hispanic or Latino patients. Compared to White individuals, the adjusted analysis found an increased odds of positivity among American Indian or Alaska Natives and Hispanic or Latinos and decreased odds among Asians, Black or African Americans (reversing associative direction from the unadjusted OR), Native Hawaiian or other Pacific Islanders. However, this reversal may have been the result of the inclusion of income and state in the adjusted model. Apart from Asian individuals, the magnitude of associations was generally small. A review of the literature found several studies which have demonstrated that COVID-19 positivity was significantly and clinically higher among most racial and ethnic minority groups, compared to White individuals (4, 14–19).

We examined patterns of new variants by race and ethnicity to understand which populations were most vulnerable to emerging disease. Exposure to new variants disproportionately exposed non-White patients to uncertain changes in virulence, transmissibility, and vaccine protection (3, 20). Although the adjusted analysis reduced the magnitude and direction of some associations, the analyses by rural/urban geography highlighted effect measure modification in the likelihood of testing positive for emerging variants by race and ethnicity. During the period where the Delta variant was emerging, Asian, Black, and African American individuals living in rural areas showed significantly higher odds of testing for Delta relative to White individuals, though the associations were not statistically significant. Racial and ethnic differences in the likelihood of Omicron differed more greatly by geography than the Delta variant. Within rural areas, Asian, Black, and African American individuals showed significantly greater likelihood of testing positive for Omicron than White individuals living in rural areas. These findings highlight the relevance of geographic location, in addition to race and ethnicity, when assessing SARS-CoV-2 related constructs and outcomes (21–23).

Although this study leveraged data from a large national pharmacy chain and implemented WGS to understand the distribution of specific SARS-CoV-2 variants, there are several limitations. First, due to the nature of testing data collection, we were unable to confirm the number of unique patients who were tested. This limited our ability to use certain statistical techniques and understand patterns in frequent testers. Second, median income was potentially misclassified as it was based on the pharmacy zip code rather than individual patients. Third, although the composition of our testing population was roughly similar to that of the US, we are unable to confirm if true testing equity was achieved. For example, as with this study, prior work shows a disproportionately greater burden of SARS-CoV-2 among non-White racial and ethnic groups. Therefore, slightly higher representation of groups with greater burden may imply true equity in access. The lack of an overrepresentation in our study may suggest persistent inequities in access and/or racial and ethnic differences in where tests were performed (e.g., in pharmacy vs. hospital settings).Additionally, home testing, which rapidly increased during the emergence of Omicron, was highest among non-white individuals (24). This likely resulted in a reduced proportion of non-white patients testing at pharmacy testing locations than would have been expected without the availability of home testing during that period. Last, Walgreens pharmacies and testing locations are distributed nationally; however, the results of this study may not generalize to other pharmacy chains and community pharmacies with different geographic distributions.

## 5 Conclusion

This study examined the relationship between patient sociodemographic characteristics, with a focus on race and ethnicity, and SARS-CoV-2 testing trends, positivity, and variant-specific epidemiology from May 2021 to February 2022 in Walgreens testing locations. When compared to the overall U.S. composition, the COVID-19 testing population was similar in race and ethnicity composition and household income. Higher SARs-CoV-2 positivity rates were observed in non-White patients. Additionally, the Omicron variant was found to be more prevalent in racial and ethnic minority groups during its emergence—especially in urban settings. Overall, this study demonstrates the potential and feasibility of pharmacy collaborations to provide SARS-CoV-2 testing access and to identify important epidemiological insights regarding racial and ethnic inequities.

## Data Availability

The data analyzed in this study is subject to the following licenses/restrictions: these data are not publicly available due to their proprietary nature. Requests to access these datasets should be directed to Tanya Singh, research@walgreens.com.
